# Forecasting Daily Patient Outflow From a Ward Having No Real-Time Clinical Data

**DOI:** 10.2196/medinform.5650

**Published:** 2016-07-21

**Authors:** Shivapratap Gopakumar, Truyen Tran, Wei Luo, Dinh Phung, Svetha Venkatesh

**Affiliations:** ^1^ Centre for Pattern Recognition and Data Analytics Deakin University Geelong Waurn Ponds Australia

**Keywords:** patient flow, discharge planning, predictive models

## Abstract

**Background:** Modeling patient flow is crucial in understanding resource demand and prioritization. We study patient outflow from an open ward in an Australian hospital, where currently bed allocation is carried out by a manager relying on past experiences and looking at demand. Automatic methods that provide a reasonable estimate of total next-day discharges can aid in efficient bed management. The challenges in building such methods lie in dealing with large amounts of discharge noise introduced by the nonlinear nature of hospital procedures, and the nonavailability of real-time clinical information in wards.

**Objective:**

Our study investigates different models to forecast the total number of next-day discharges from an open ward having no real-time clinical data.

**Methods:**

We compared 5 popular regression algorithms to model total next-day discharges: (1) autoregressive integrated moving average (ARIMA), (2) the autoregressive moving average with exogenous variables (ARMAX), (3) k-nearest neighbor regression, (4) random forest regression, and (5) support vector regression. Although the autoregressive integrated moving average model relied on past 3-month discharges, nearest neighbor forecasting used median of similar discharges in the past in estimating next-day discharge. In addition, the ARMAX model used the day of the week and number of patients currently in ward as exogenous variables. For the random forest and support vector regression models, we designed a predictor set of 20 patient features and 88 ward-level features.

**Results:**

Our data consisted of 12,141 patient visits over 1826 days. Forecasting quality was measured using mean forecast error, mean absolute error, symmetric mean absolute percentage error, and root mean square error. When compared with a moving average prediction model, all 5 models demonstrated superior performance with the random forests achieving 22.7% improvement in mean absolute error, for all days in the year 2014.

**Conclusions:**

In the absence of clinical information, our study recommends using patient-level and ward-level data in predicting next-day discharges. Random forest and support vector regression models are able to use all available features from such data, resulting in superior performance over traditional autoregressive methods. An intelligent estimate of available beds in wards plays a crucial role in relieving access block in emergency departments.

## Introduction

Demand for health care services has become unsustainable [1,2]. This is largely due to increase in population and life expectancy, escalating costs, increased patient expectations, and workforce issues [3]. Despite increased demands, the number of inpatient beds in hospitals has come down by 2% since the last decade [2,4]. Efficient bed management is key to meeting this rising demand and reducing health care costs.

Daily discharge rate can be a potential real-time indicator of operational efficiency [5]. From a ward-level perspective, a good estimate of next-day discharges will enable hospital staff to foresee potential problems such as changes in number of available beds and changes in number of required staff. Efficient forecasting reduces bed crisis and improves resource allocation. This foresight can help accelerate discharge preparation, which has huge cost on clinical staff and educating patients and family, requiring postdischarge planning [6,7]. However, studying patient flow from general wards offers several challenges.

Ward-level discharges incorporate far greater hospital dynamics that are often nonlinear [8]. Accessing real-time clinical information in wards can be difficult because of administrative and procedural barriers, such data may not be available for predictive applications. Because the diagnosis coding is performed after discharge, there is little information about medical condition or variation in care quality in real time. In addition, factors other than patient condition play a role in discharge decisions [5,9,10].

**Figure 1:**
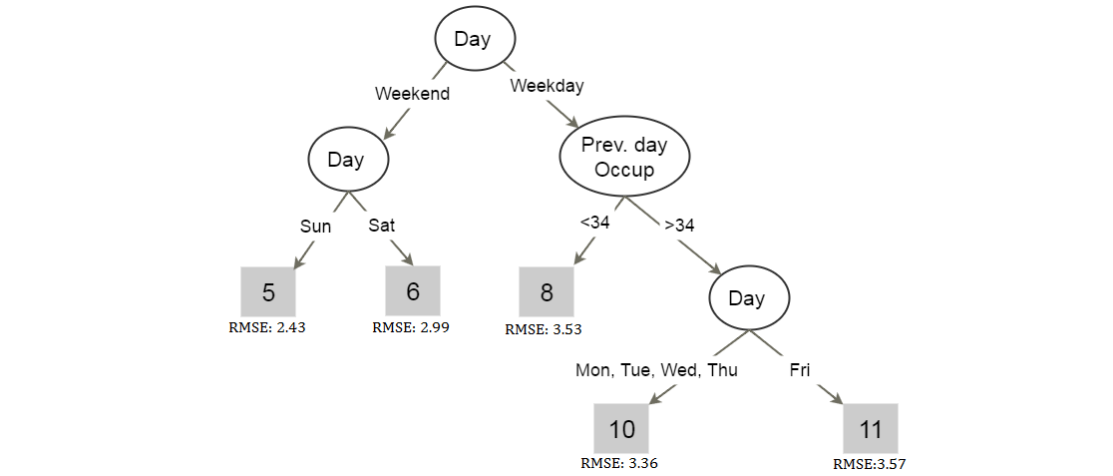
Decision tree modeling of total discharges from an open ward from day of the week and ward occupancy (previous day occupation) data for 5 years. The leaves represent total number of patient discharges.

The current practice of bed allocation in general wards of most hospitals involves a hospital staff/team, who use past information and experience, to schedule and assign beds [11]. Modern machine learning techniques can be used to aid such decisions and help understand the underlying process. As an example, Figure 1 illustrates a decision tree trained on past discharges and ward occupancy statistics, which models the daily discharge pattern from an open ward in a regional Australian hospital. Although the absence of patient medical information affected forecast performance, the decision rules provide important insight into the discharge process.

Motivated by this result, we address the open problem of forecasting daily discharges from a ward with no real-time clinical data. Specifically, we compare the forecasting performance of 5 popular regression models: (1) the classical autoregressive integrated moving average (ARIMA), (2) the autoregressive moving average with exogenous variables (ARMAX), (3) k-nearest neighbor (kNN) regression, (4) random forest (RF) regression, and (v) support vector regression (SVR). Our experiments were conducted on commonly available data from a recovery ward (heath wing 5) in Barwon Health, a regional hospital in Victoria, Australia. The ARIMA and kNN models are built from daily discharges from ward. To account for the seasonal nature of discharges, the ARMAX model included day of the week and ward occupancy statistics. We identified and constructed 20 ward-level and 88 patient-level predictors to derive the RF and SVR models.

Forecasting accuracy was measured using 3 metrics on a held out set of 2511 patient visits in the year 2014. When compared with a naive forecasting method of using the mean of last week discharges, we demonstrate through our experiments that (1) using regression methods for forecasting discharge outperforms naive forecasting, (2) SVR and RF models outperform the autoregressive methods and kNN, (3) an RF model derived from 108 features has the minimum error for next-day forecasts.

The significance of our study is in identifying the importance of foreseeing available beds in wards, which could help relieve emergency access block [12].

Patient length of stay directly contributes to hospital costs and resource allocation. Long-term forecasting in health care aims to model bed and staffing needs over a period of months to years. Cote and Tucker categorize the common methods in health care demand forecasting as percent adjustment, 12-month moving average, trendline, and seasonalized forecast [13]. Although each of these methods is built from historical demand, seasonalized forecasting provides more realistic results as it takes into account the seasonal variations and trends in the data. Mackay and Lee [3] advise modeling the patient flow in health care institutions for tactical and strategic forecasting. To this end, compartmental modeling [14,15], queuing models [16,17] and simulation models [17-20] have been applied to analyze patient flow. To understand long-term patient flow, studies analyze metrics such as bed occupancy [3,8,14,19,21,22], patient arrivals [23], and individual patient length of stay [19,24-27].

On the other hand, our work implements short-term forecasting. The short-term forecasting methods are concerned with hourly and daily forecasts from a single unit in a care environment. The most popular unit of interest is the emergency or acute care department because this is often a key performance indicator metric in assessing quality of care [28,29].

### Time Series and Smoothing Methods

When looking at discharges as time series, autoregressive moving average models are the most popular [30-32]. Exponential smoothing techniques have also been used to forecast monthly [33] and daily patient flow [34].

Jones et al used the classical ARIMA to forecast daily bed occupancy in emergency department of a European hospital [30]. The model which included seasonality terms demonstrated reasonable performance to predict bed occupancy. The authors speculated whether nonlinear forecasting techniques could improve over ARIMA. A recent study confirmed the effectiveness of this forecasting technique in a US hospital setting [35]. ARIMA models were also successfully used to forecast the number of occupied beds during a SARS outbreak in a Singapore hospital [36]. A recent study used patient attendances in a pediatric emergency department to model daily demand using ARIMA [37].

Jones et al [34] compared the ARIMA mode with exponential smoothing and artificial neural networks to forecast daily patient volumes in emergency department. The study revealed no single model to be superior and concluded that seasonal patterns play a major role in daily demand.

### Simulation Methods

Modeling using simulation is typically used to study the behavior of complex systems. An early work in investigated the effects of emergency admissions on daily bed requirements in acute care, using discrete-event stochastic simulation modeling [38]. Sinreich and Marmor [39] proposed a guide for building a simulation tool based on data from emergency departments of 5 Israeli hospitals. Their method analyzed the flow of patients clustered into 8 types along with time elements. The simulation demonstrated that patient processes are better characterized by type of the patients, rather than specific hospitals visited. Yeh and Lin used a simulation model to characterize patient flow through a hospital emergency department and reduced waiting times using a genetic algorithm [40]. A similar experiment was carried out in a geriatric department using a combination of discrete event simulation and queuing model to analyze bed occupancy [19].

### Regression for Forecasting

Regression models analyze the relationship between the forecasted variable and features in the data. Linear regression that encoded monthly variations was used to forecast patient admissions over a 6-month horizon and outperformed quadratic and autoregressive models [41]. Another study used clustering and Principle Component Analysis PCA to find significant predictors from patient data to model emergency length of stay using linear regression [42]. A nonlinear approach using regression trees was proposed in forecasting patient admissions which demonstrated superior performance over a neural net framework [43].

Barnes et al used 10 predictors to model real-time inpatient length of stay in a 36-bed unit using an RF model [24].

Nonlinear regression is better suited to model the changing dynamics of patient flow. To characterize the outflow of patients from the ward, we resort to regression using RF, kNN, and SVR. In the area of pattern recognition, kNNs [44] are the most effective method that exploits repeated patterns. The kNN algorithm has been successfully applied to forecast to histogram time series in financial data [45]. The nonparametric regression using kNN has been successfully demonstrated for short-term traffic forecasting [46,47] and electricity load forecasting [48,49]. However, kNN regression has not been studied for patient flow.

Another powerful and popular regression technique, SVR, uses kernel functions to map features into a higher dimensional space to perform linear regression. Though this technique has not seen much application in medical forecasting, support vector machines have been successful in financial market prediction, electricity forecasting, business forecasting, and reliability forecasting [50].

Apart from the standard autoregressive methods, we use kNN, RFs, and SVR in forecasting next-day discharges. Because discharge patterns repeat over time, kNN regression can be applied to search for a matching pattern from past discharges. RFs and SVR regression are powerful modelling techniques requiring minimum tuning to effectively handle nonlinearity in the hospital processes.

Recently, RF forecasting was used to predict total patient discharges from a 36 bed unit in an urban hospital [24]. Apart from 4 demographic and 2 timing predictors, this study used 3 clinical predictors for patients: (1) reason for visit: identified by a physician and recorded using International Classification of Diseases: version 9 (ICD-9) diagnosis codes [51], (2) observation status: assigned to patients for monitoring purpose, and (3) pending discharge location. Total number of discharges was estimated from aggregate of individual patient length of stay.

The absence of real-time clinical information in our data makes calculating patient length of stay impossible. Instead, we resort to modelling next-day discharges by observing previous discharge patterns and examining demographics and flow characteristics in the ward.

## Methods

### Data

Our study used retrospective data collected from a recovery ward in Barwon Health, a large public health provider in Victoria, Australia serving about 350,000 residents. Ethics approval was obtained from the Hospital and Research Ethics Committee at Barwon Health (number 12/83) and Deakin University. The total number of available beds depended on the number of staff assigned to the ward. On average, the ward had 36 staffed beds, but fluctuated between 20 and 80 beds with varying patient flow. The physicians in the ward had no teaching responsibilities.

**Table 1:** Tables in hospital database used in our data collection.

Tables	Columns
Patients	1. Patient ID
	2. Age
	3. Gender
Ward Stay	1. Admission ID
	2. Name of the ward
	3. Time (entry, exit)
	4. Bed ID
Admissions	1. Patient ID
	2. Admission ID
	3. Time (admit, discharge)
	4. Patient Class (21 categories)
	5. Admission type (7 categories)

**Table 2:** Cohort details.

Cohort	Stats
Total patient visits	12,141
Unique patients	10,610
Length of stay (mean, median, IQR^a^)	4.26, 3, 5
Discharges per day (mean, median, IQR)	8.7, 8, 5
Admissions per day (mean, median, IQR)	8.6, 8, 5
Mean ward occupancy, IQR	30.9, 4
Gender	54.8% Female
Age (mean, median)	66, 63.23

^a^IQR, interquartile range.

The data for our study came from three tables in the hospital database, as shown in Table 1. Additional real-time data that described patient condition or disease progression were unavailable because diagnosis coding using medical codes is done after discharge. Patient flow was collected for a period of 4 years. Using the admission and discharge times for each patient, we calculated the daily discharges from our ward in study. A total of 12,141 patients were admitted into the ward with a median discharge of 8 patients per day from January 1, 2010, to December 31, 2014. Table 2 summarizes the main characteristics of our data.

**Figure 2:**
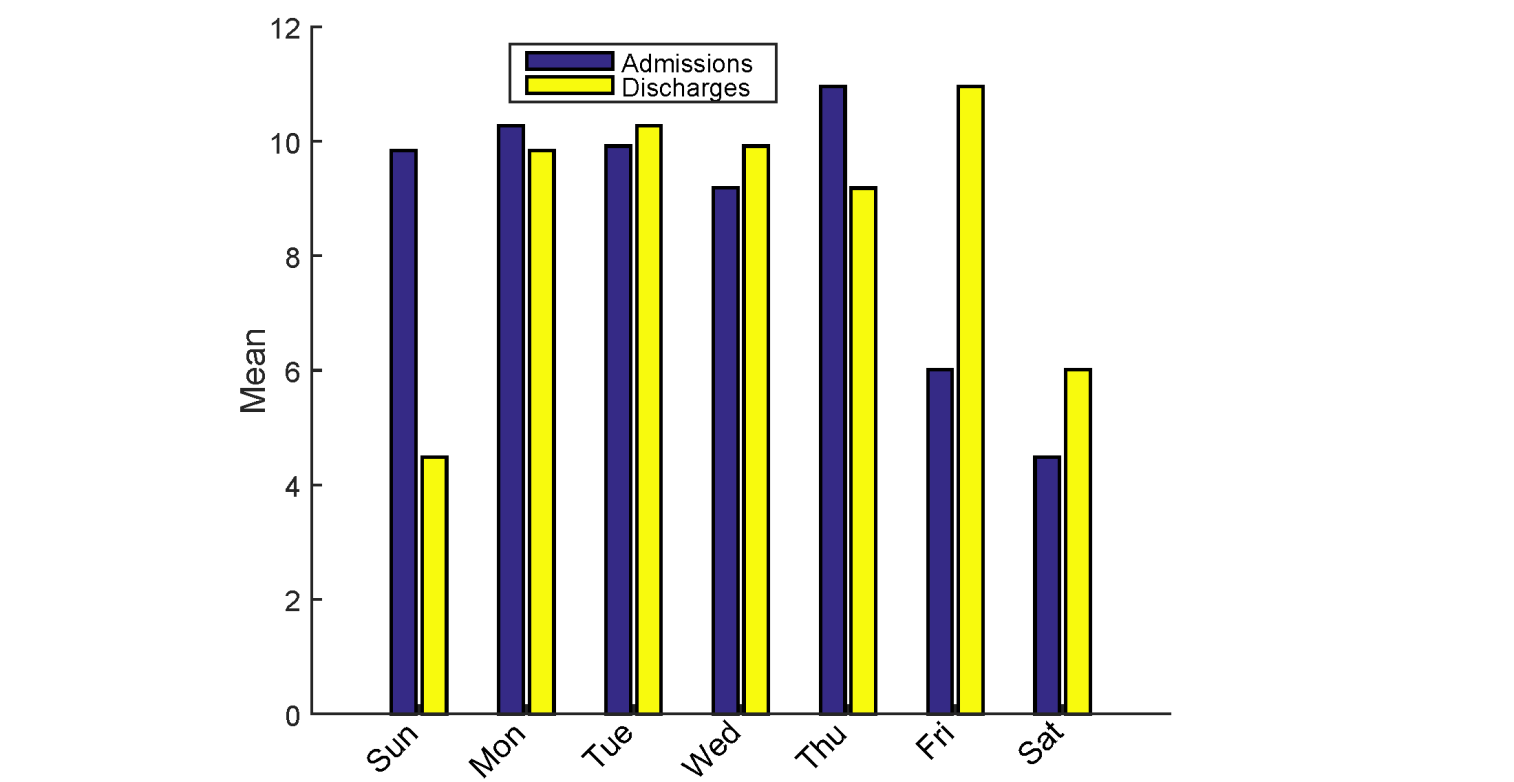
Mean admissions and discharges per day from ward.

**Figure 3:**
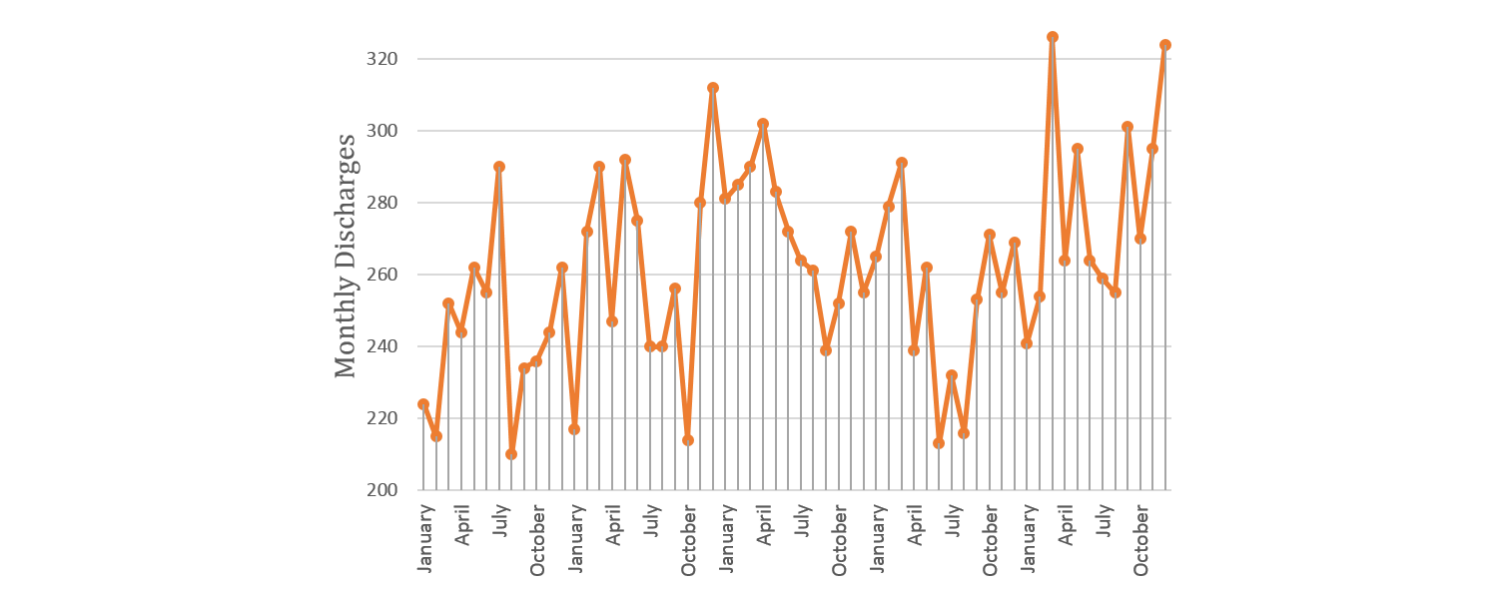
Time series of monthly discharges from ward.

A time series decomposition of our data revealed strong seasonal variations and high nonlinearity in daily discharge patterns. There was a defined weekly pattern–discharge from ward peaked on Fridays and dropped significantly on weekends (see Figure 2). This seasonal nature is in tune with previous studies [9,32]. Aggregating the daily discharges into a monthly time series revealed defined monthly patterns (see Figure 3). The data displayed no significant trend. In addition, the daily discharge pattern was found to be highly nonlinear. Our forecasting methods must be able to handle such data dynamics.

We describe the following diverse methods that are applicable to forecasting under complex data dynamics: (1) ARIMA, (2) autoregressive moving, (3) forecasting using kNN discharge patterns, (4) RF, and (5) SVR. Autoregressive methods model the temporal linear correlation between nearby data points in the time series. Nearest patterns lift this linearity assumption and assumes that short periods form repeated patterns. Finally, RF and SVR look for a nonlinear functional relationship between the future outcomes and descriptors in the past.

### Forecasting Methods

#### Autoregressive Integrated Moving Average

**Figure 4:**
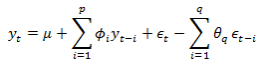
Classical ARIMA model.

Time-series forecasting methods can analyze the pattern of past discharges and formulate a forecasting model from underlying temporal relationships [52]. Such models can then be used to extrapolate the discharge time series into the future. ARIMA models are widely used in time-series forecasting. Their popularity can be attributed to ease of model formulation and interpretability [53]. ARIMA models look for linear relationships in the discharge sequence to detect local trends and seasonality. However, such relationships can change over time. ARIMA models are able to capture these changes and update themselves accordingly. This is done by combining autoregressive (AR) and moving average (MA) models. Autoregressive models formulate discharge at time *t*=y_t_, as a linear combination of previous discharges. On the other hand, moving averages models characterize as linear combination of previous forecast errors. For ARIMA model, the discharge time series is made stationary using differencing. Let *∅* be autoregressive parameters, *θ* be moving average parameters, and *ϵ* be the forecast errors. Such an ARIMA model can be defined as shown in Figure 4, where *µ* is a constant. By varying *p* and *q*, we can generate different models to fit the data. Box Jenkins method [54] provides a well-defined approach for model identification and parameter estimation. In our work, we choose the auto.arima() function from the forecast package [55] in R [56] to automatically select the best model.

#### Autoregressive Moving Average With Exogenous Variables (ARMAX)

**Figure 5:**
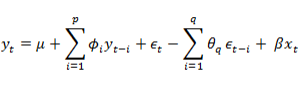
ARIMA model with exogenous variable xt.

Dynamic regression techniques allow adding additional explanatory variables, like day of the week and number of current patients in the ward, to autoregressive models. The autoregressive moving ARMAX modifies ARIMA model by including depending external variable *x*_t_ at time *t*, as shown in Figure 5. We model *x*_t_ using features from the hospital database.

#### Detecting Discharge Patterns Using k-Nearest Neighbors

**Figure 6:**
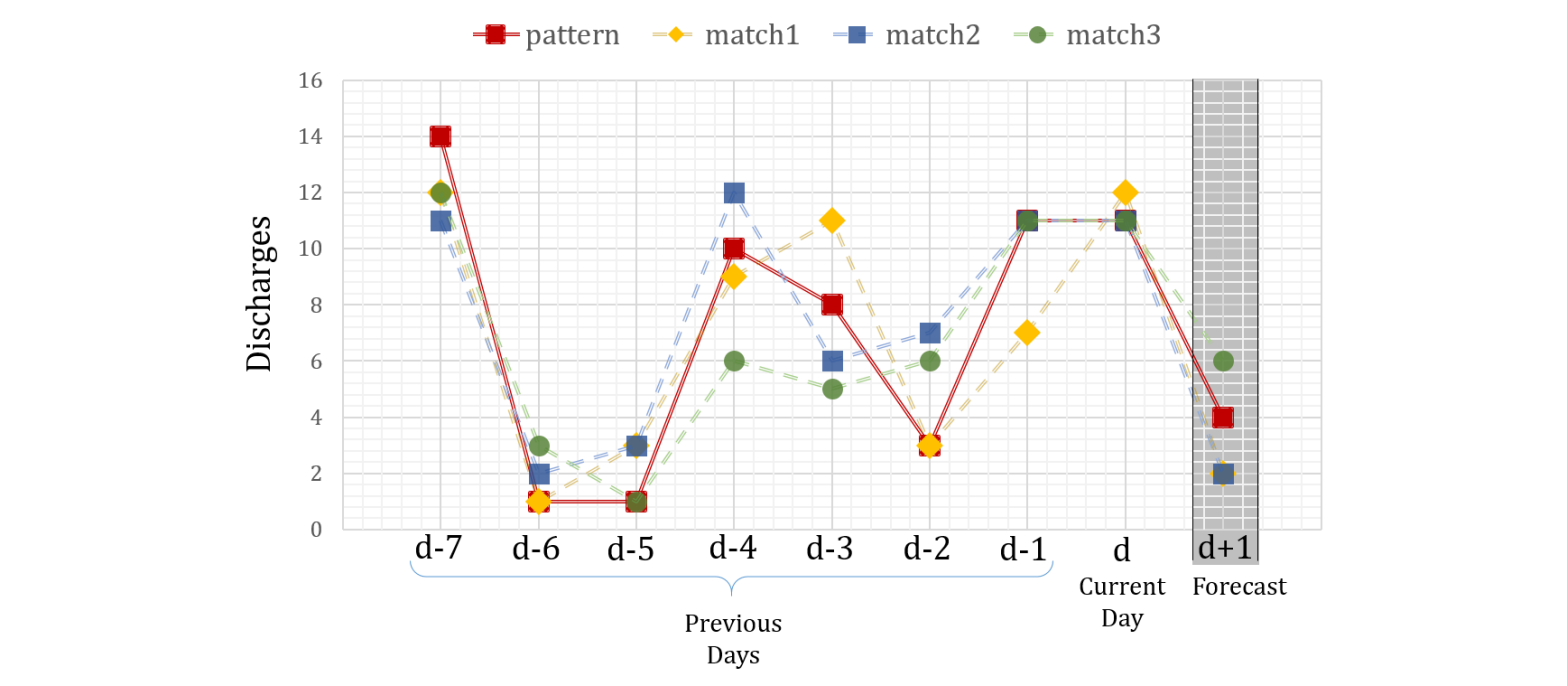
k-nearest neighbor forecasting example with k=3 and *P*=7.

The kNN algorithm takes advantage of the locality in data space. We assume that the next-day discharge depends on the discharges happening in previous days. Using kNN principles, we can do a regression to forecast the next-day discharge. Let *y*_d_ represent number of discharges on the current day: *d*. To forecast the next day discharge: *y*_d+1_, we look at the discharges over the past *p* days as: disch_vec=[*y*_d-p_: *y*_d_]. Using Euclidean distance metric, we find *k* closest matches to disch_vec from the training data. An estimate of next-day discharge: *ŷ*_d+1_, is calculated as a measure of the next-day discharges of the *k* matched patterns: (*y*_match_)_i_*i* ϵ(1:k). Figure 6 shows an example of kNN based forecasting. Here, disch_vec in red [*y*_d-7_: *y*_d_] results in 3 matches from the training data. For simplicity, we have plotted the matched patterns alongside disch_vec, although they had occurred in the past. The next-day forecast *ŷ*_d+1_ becomes a measure of (*y*_match_)_i_, where (*y*_match_)_i_*i ϵ* (1:3) is the (*d* +1)^th^term of each of the matched patterns [57].

**Figure 7:**
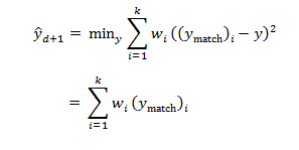
Calculating ŷd+1 by minimizing the weighted quadratic loss.

One popular method of calculating *ŷ*_d+1_ is by minimizing the weighted quadratic loss (Figure 7), where *w*_i_ takes values between 0 and 1, with ∑^k^_i=1_*w*_i_*=1*_._ However, there are 2 main drawbacks making it less desirable for our data. First, the quadratic loss is sensitive to outliers. Second, a robust estimate of { *w*_i_} becomes difficult.

**Figure 8:**
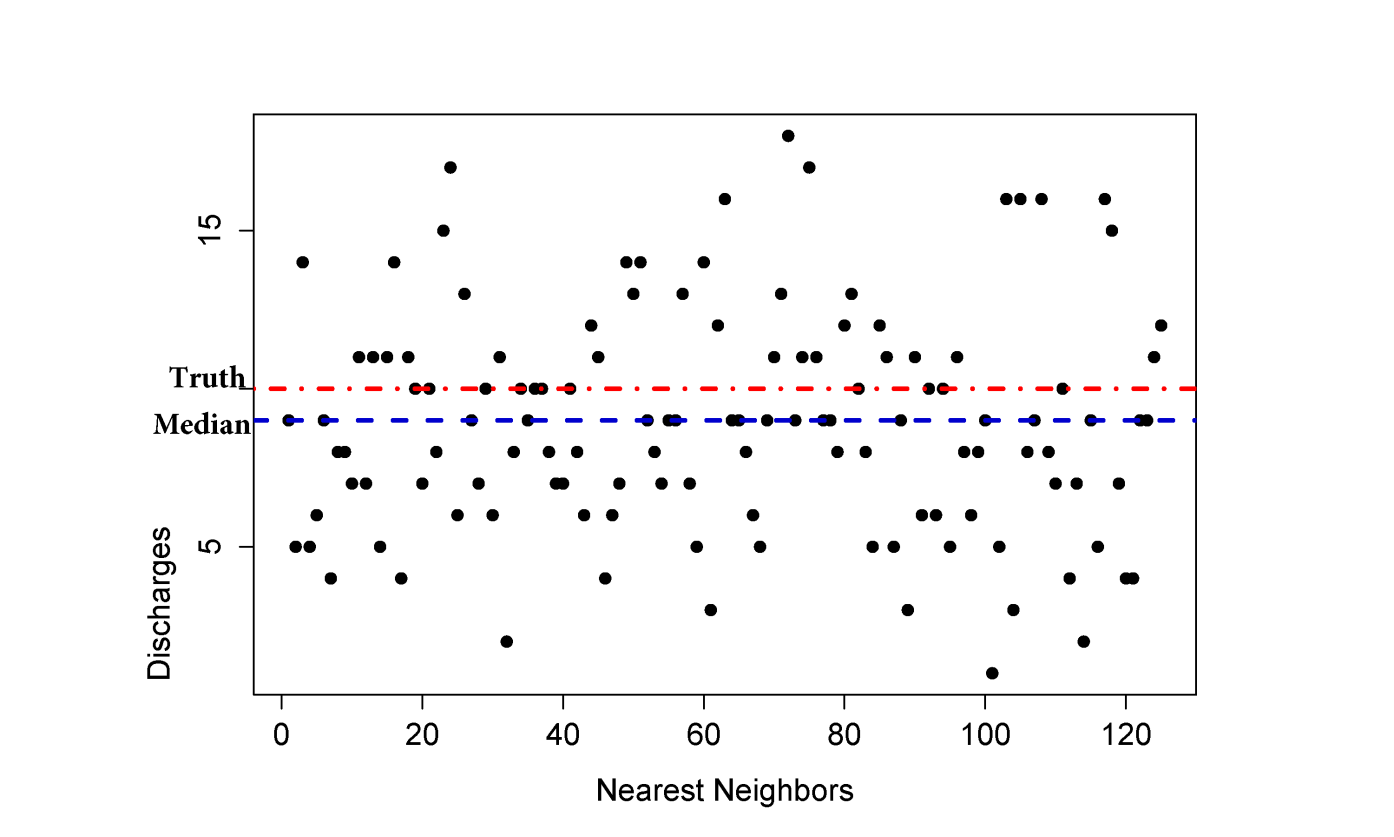
Scatterplot of next-day forecast using k-nearest neighbor for a given day. X-axis represents each matched nearest-neighbor pattern. Y-axis represents the next day forecast of that matched pattern.

**Figure 9:**
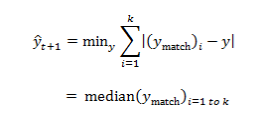
Estimating ŷt+1 by minimizing the robust loss.

Our data contain significant noise, causing large variations in next-day forecasts of the *k* matched patterns. We illustrate this problem in Figure 8. For a given day, kNN regression returns 125 matched patterns. The next-day forecasts from each k=125 patterns displayed significant variations. In such scenario, we resort to estimating *ŷ*_t+1_ by minimizing the robust loss (Figure 9).

#### Random Forest

**Figure 10:**
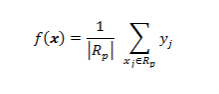
Random forests formulation of next day discharges (y) from historical descriptors (x).

In this approach, we assume the next-day discharge as a function of historical descriptor vector: *x*. We use each day in the past as a data point, where the next-day discharge is the outcome, and the short period before the discharge are used to derive descriptors. The RF used in this paper is currently one of the most powerful methods to model the function *y*= *f* (*x*) [58,59]. An RF is an ensemble of regression trees. A regression tree approximates a function *f* (*x*) by recursively partitioning the descriptor space. At each region *R*_p_, the function is approximated as shown in Figure 10, where | *R*_p_| is the number of data point falling in region *R*_p_*.* The RF creates a diverse collection of random trees by varying the subsets of data points to train the trees and the subsets of descriptors at each step of space partitioning. The final outcome of RF is an average of all trees in the ensemble. Since tree growing is a highly adaptive process, it can discover any nonlinear function to any degree of approximation if given enough training data. However, the flexibility makes regression tree prone to overfitting, that is, the inability to generalize to unseen data. This requires controlling the growth by setting the number of descriptors per partitioning step, and the minimum size of region *R*_p_*.*

The voting leads to great benefits: reduce the variations per tree. The randomness helps combat against overfitting. There is no assumption about the distribution of data or the form of the function (*x*). There is controllable quality of fits.

#### Support Vector Regression

**Figure 11:**
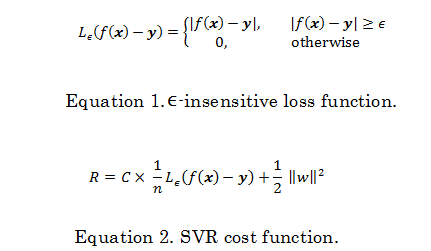
The SVR learning model.

The historical descriptor vector *x,* used in the RF model can also be used to build a SVR model [60]. Given the set of data {(*x*_1_, *y*_1_), (*x*_2_, *y*_2_), … (*x*_n_, *y*_n_)}, where each *x*_i_ϵ *R*^m^ denotes the input descriptor for the corresponding next day forecast *y*_i_ϵ *R*^1^, a regression function takes the form: *ŷ*_i_= *f* (*x*_i_). SVR works by (1) mapping the input space of *x*_i_ into a higher dimensional space using a nonlinear mapping function: *ϕ*, (2) performing a linear regression in this higher dimensional space. In general, we can express the regression function as: *f* (*x*) = (*wϕ* (*x*))+ *b,* where, *w* ϵ *R*^m^ is the weights and *b* ϵ *R*^1^is the bias term. Vapnik [60] proposed the ϵ-insensitive loss function for SVR, which takes the form as shown in Equation 1 in Figure 11. The loss function *L*_ϵ_ tolerates errors that are smaller than the threshold: *ϵ,* resulting in a “tube” around the true discharge values. Model parameters can be estimated by minimizing the cost function as shown in Equation 2 in Figure 11, where *C* is a constant that penalizes error in training data.

In our work, we use an RBF kernel [61] for mapping our input data to higher dimensional feature space. RBF kernels are a good choice for fitting our nonlinear discharge pattern because of its ability to map the training data to an infinite dimensional space and easy implementation. The solution to the dual formulation of SVR cost function is detailed in [60,62].

### Experiments

**Figure 12:**
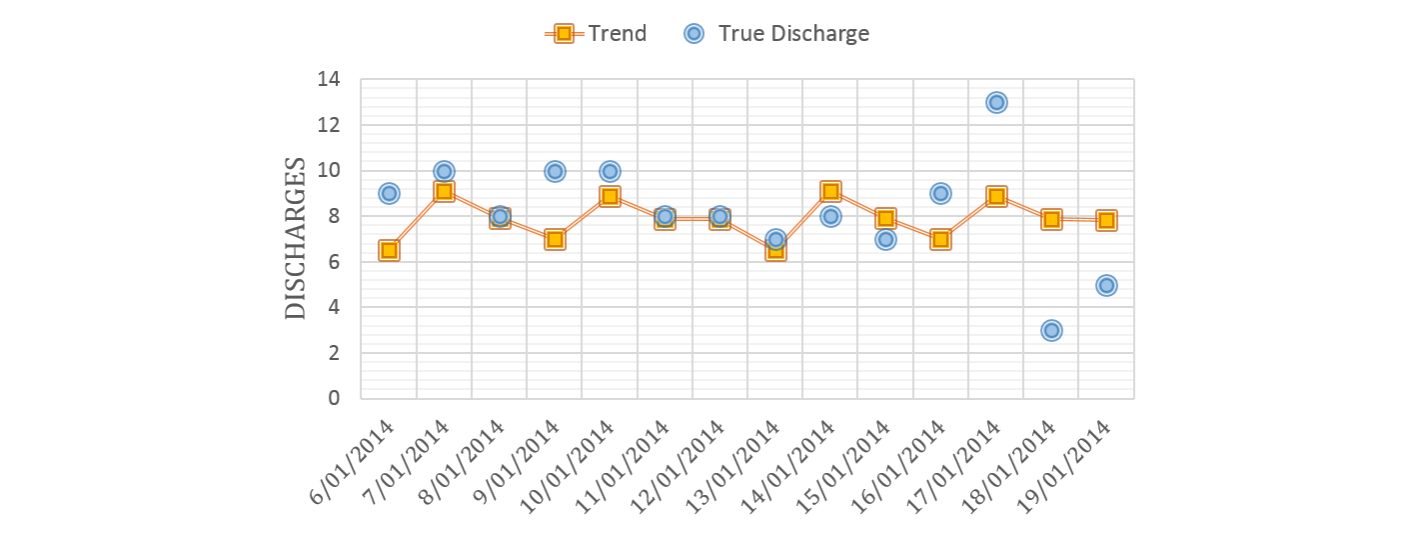
An example of the discharge trend, as derived from a locally weighted polynomial regression model.

We extracted all data from the database tables (as in Table 1) for our ward in study. Patient flow was analyzed for a period of 5 years. We formatted our data as a matrix where each row corresponds to a day and each column represents a feature (descriptor). Two main groups of features were identified: (1) ward level and (2) patient level. Our feature creation process resulted in 20 ward-level and 88 patient-level predictors, as listed in Table 3. The ward-level descriptor: trend of next-day discharge was calculated by fitting a locally weighted polynomial regression [63] from past discharges. An example of this regression fitting is shown in Figure 12.

**Table 3:** Features constructed from ward data in hospital database.^a^

Type	Predictor	Description
Ward-level	Seasonality	Current day-of-week, current month
	Trend	Calculated using locally weighted polynomial regression from past discharges on the same weekday
	Admissions	Number of admissions during past 7 days
	Discharges	Number of discharges during past 7 days, number of discharges in previous 14th day and 21st day
	Occupancy	Ward occupancy in previous day
		
Patient-level	Admission type	5 categories
	Patient referral	49 categories
	Patient class	21 categories
	Age category	8 categories
	Number of wards visited	4 categories
	Elapsed length of stay	Calculated daily for each patient in the ward

^a^ The random forest and support vector regression models used the full set of features. The ARMAX (autoregressive moving average with exogenous variables) model used seasonality and occupancy. All other models were derived from daily discharges.

### Evaluation Protocol

Our training and testing sets are separated by time. This strategy reflects the common practice of training the model using data in the past and applying it on future data. Training data consisted of 1460 days from January 1, 2010, to December 31, 2013. Testing data consisted of 365 days in the year 2014. The characteristics of the training and validation cohort are shown in Table 4. Most stays were short, around 65% of patients stayed for less than 5 days.

**Table 4:** Characteristics of training and validation cohorts.

Categorization		Training (2010-2013)	Testing (2014)
Total days		1460	365
Mean discharges per day		8.47	9.17
Number of admissions		9630	2511
Gender			
	Male	4329 (44.9%)	1135 (45.2%)
	Female	5301 (55.1%)	1376 (54.8%)
Mean age (years)		63.65	61.62
Length of stay			
	1-4 days	6377 (66.22%)	1636 (65.15%)
	5 or more days	3253 (33.78%)	875 (34.85%)

#### Baseline Forecasting

The current hospital strategy involves using past experience to foresee available beds. To compare the efficiency of our proposed approaches, we model the following baselines: (1) Naive forecasting using the last day of week discharge: since our data were found to have defined weekly patterns, we model the next day discharge as the number of discharges for the same day during previous week; (2) naive forecasting using mean of last week discharges: to better model the variation and noise in weekly discharges, we model the next-day discharge as the mean of discharges during previous 7 days; and (3) naive forecasting using mean of last 3-week discharges: to account for the monthly and weekly variations in our data, we use mean of daily discharges over the past 3 weeks to model the next-day discharge.

#### Measuring Forecast Performance

We compare the next-day forecasts of our proposed approaches with the baseline methods on the measures of mean forecast error, mean absolute error, symmetric mean absolute percentage error, and root mean square error [64,65]. If *y*_t_ is the measured discharge at time *t*, *f*_t_ is the forecasted dishcharge at time *t*, we can define the following:

• Mean forecast error (MFE): is used to gauge model bias and is calculated as MFE = mean(*y*_t_- *f*_t_)

• For an ideal model, MFE = 0. If MFE > 0, the model tends to underforecast. When MFE < 0, the model tends to overforecast.

• Mean absolute error (MAE): is the average of unsigned errors: MAE = mean| *y*_t_- *f*_t_|.

MAE indicates the absolute size of the errors.

• Root mean square error (RMSE) is a measure of the deviation of forecast errors. It is calculated as: RMSE = √mean(*y*_t_- *f*_t_*)*^2^

Due to squaring and averaging, large errors tend to have more influence over RMSE. In contrast, individual errors are weighted equally in MAE. There has been much debate on the choice of MAE or RMSE as an indicator of model performance [66,67].

•Symmetric mean absolute percentage error (sMAPE): It is scale independent and hence can be used to compare forecast performance between different data series. It overcomes 2 disadvantages of mean absolute percentage error (MAPE) namely, (1) the inability to calculate error when the true discharge is zero and (2) heavier penalties for positive errors than negative errors. sMAPE is a more robust estimate of forecast error and is calculated as: sMAPE = mean(200[| *y*_t_- *f*_t_|/ *y*_t_+ *f*_t_]). However, sMAPE ranges from −200% to 200%, giving it an ambiguous interpretation [68].

## Results

### Model Performance

In this section, we describe the results of comparing our different forecasting methods. The model parameters for kNN forecast, RF, and SVR models were tuned to minimize forecast errors.

For kNN regression, the optimum value of pattern length: *d* and number of nearest neighbours: *k*, was obtained by analyzing forecast RMSE for values *d* ϵ (1,100) and *k* ϵ(5,1000). Minimum RMSE of 3.77 was obtained at *d*=70 and *k*=125.

The SVR parameters *C* (penalty cost) and *ϵ* (amount of allowed error) were determined by choosing the best value from a grid search, that minimized the model RMSE. Similarly, the optimum number of variables in building each node of the RF was chosen by examining its effect on minimizing the out-of-bag estimate.

**Figure 13:**
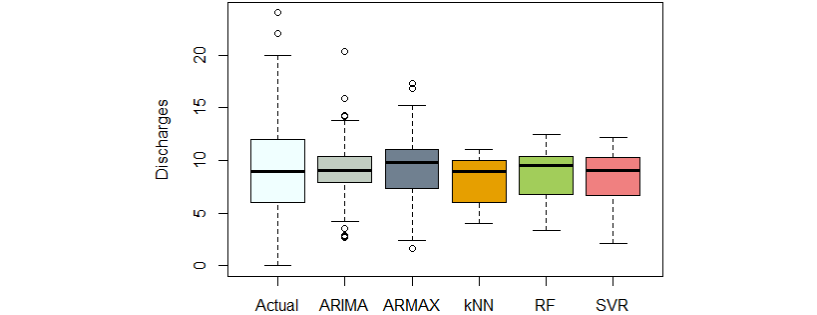
Comparison of actual and forecasted discharges from ward for each day in 2014.

We compared the naive forecasting methods with our proposed 5 models using MFE, MAE, RMSE, and sMAPE. The results are summarized in Table 5, whereas Figure 13 compares the distribution of actual discharges with different model forecasts.

**Table 5:** Forecast accuracy of different models.

Model		Mean forecast error	Mean absolute error	Symmetric mean absolute percentage error	Root mean square error	Mean absolute error improve over naïve
Naive forecast						
	Using discharge from last weekday	0.03	3.81	45.70 %	4.95	
	Using mean of last week discharges	0.02	3.57	41.68 %	4.42	
	Using mean of last 3-week discharges	0.04	3.44	40.14%	4.34	
ARIMA^a^		0.06	3.27	38.32 %	4.15	4.9 %
ARMAX^b^		-0.01	2.99	34.86 %	3.84	13.1 %
k-nearest neighbor		1.09	2.88	34.92 %	3.77	16.3 %
Support vector regression		0.73	2.75	32.88%	3.64	20.1 %
Random forest		0.44	2.66	31.86 %	3.49	22.7 %

^a^ ARIMA: autoregressive integrated moving average

^b^ ARMAX: autoregressive moving average with exogenous variables

The naive forecasts are unable to capture all variations in the data and resulted in the maximum error when compared with other models.

The variations in seasonality and trend are better captured in ARIMA and ARMAX models. The time series consisting of past 3-month discharges were used to generate the next-day discharge forecast. The ARMAX model also included the day of week and ward occupancy as exogenous variables, which resulted in better forecast performance over ARIMA.

**Figure 14:**
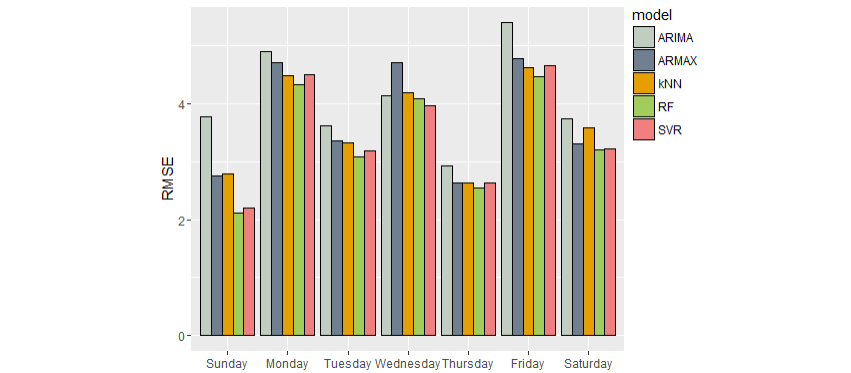
Forecast error in predicting each day of week in 2014.

Interestingly, kNN was more successful than ARIMA and ARMAX in capturing the variations in discharge, demonstrating about 3% improvement in MAE, when compared with ARMAX. However, the kNN model tends to under forecast (MFE = 1.09), possibly because of resorting to median values for forecast. In comparison, RF and SVR forecast models demonstrated better performance. This can be expected because they are derived from all the 108 features. However, RF demonstrated a relative improvement of 3.3 % in MAE over SVR model (see Table 5). When looking at forecast errors for each day of week, RF model confirmed better performance, as shown in Figure 14.

The process of SVR with RBF kernel maps all data into a higher dimensional space. Hence, the original features responsible for forecast cannot be recovered, and the model acts as a black box. Alternatively, RF algorithm returns an estimate of importance for each variable for regression. Examining the features with high importance could give us a better understanding of the discharge process.

### Feature Importance in the Random Forest model

The features in random forecast model were ranked on importance scores. The top 10 significant features are described as follows. The day of week for the forecast proved to be the most important feature. Other features were number of patients in the ward during the day of forecast, the trend of discharges measured using locally weighted polynomial regression, number of discharges in past 14th day, number of discharges in past 21st day, number of patients who had visited only one previous ward, the number of males in the ward, number of patients labelled as: “public standard,” and current month of forecast.

## Discussion

### Principal Findings

Improved patient flow and efficient bed management is key to counter escalating service and economic pressures in hospitals. Predicting next-day discharges is crucial but has been seldom studied for general wards. When compared with emergency and acute care wards, predicting next-day discharges from a general ward is more challenging because of the nonavailability of real-time clinical information. The daily discharge pattern is seasonal and irregular. This could be attributed to management of hospital processes such as ward rounds, inpatient tests, and medication. The nonlinear nature of these processes contributes to unpredictable length of stay even in patients with similar diagnosis.

Typically, for open wards, a floor manager uses previous experience to foresee the number of available beds. In this paper, we attempt to model total number of next-day discharges using 5 methods. We have compared the forecasting performance using MAE, RMSE, and sMAPE. Our predictors are extracted from commonly available data in the hospital database. Although the kNN method is simple to implement, requiring no special expertise, software packages for other models are available for all common platforms. These models can be implemented by the analytics staff in hospital IT department and can be easily integrated into existing health information systems.

In our experiments, forecast based on RF model outperformed all other models. Forecasting error rate is 31.9% (as measured by sMAPE) which is in the same ballpark as the recent work of [24], though we had no real-time clinical information. An RF model makes minimum assumptions about the underlying data. Hence, it is the most flexible, and at the same time, comes with great overfitting control. Similarly, SVR also demonstrated superior performance, compared with the autoregressive and kNN models. The RBF kernel maps the features into a higher dimensional space during the regression process. Hence, the physical meaning of the features is lost, making it difficult to interpret the model. Finally, RFs and SVR are able to handle more features. This extra information in the form of patient demographics and past admission and discharge statistics contributed to improve the predictive performance when compared with other models.

The kNN regression also performed well as it assumes only the locality in the data. But it is not adaptive, and thus less flexible in capturing complex patterns. The kNN regression assumes similar patterns in past discharges extrapolate to similar future discharge, which is not true for daily discharges from ward. ARMAX model outperformed the traditional ARIMA forecasts since it incorporated seasonal information as external regressors. As expected, a naive forecast of using the median of past discharges performed worst.

We noticed a weekly pattern (Figure 2) and monthly pattern (Figure 3) in discharges from the ward. Other studies have also confirmed that discharges peak on Friday and drop during weekends [5,9,10]. This “weekend effect” could be attributed to shortages in staffing or reduced availability of services like sophisticated tests and procedures [10,69]. This suggests discharges are heavily influenced by administrative reasons and staffing.

Feature importance score from an RF model helps in identifying the features contributing to the regression process. The day of forecast proved to be one of the most important features in the RF model. Other important features included trend based on nonlinear regression of past weekdays, number of discharges in the past days, ward occupancy in previous day, number of males in the ward, and number of general patients in ward.

When looking at for each day of the week, the RF and SVR model consistently outperformed other models. Sundays and Thursdays proved to be the easiest to predict for all models (Figure 14). This can be expected since these days had the least variation in our data. Fridays proved to be the most difficult to forecast. Retraining the RF model by omitting “day of the week” increased the forecast error by 1.39% (as measured by sMAPE).

Patient length of stay is inherently variable, partly due to the complex nonlinear structure of medical care [8]. The number of discharges from a ward is strongly related to the length of stay of the current patients in the ward. Hence, the variability in ward-level discharges is compounded by the variability in individual patient length of stay. In our study, the daily discharge pattern from ward shows great variation for each day of week. Apart from patient level details, we believe that a knowledge of hospital policies is also required to capture such nonlinearity.

### Practical Significance

In our study, we were able to validate that the weekend patterns affect discharges from a general ward. The RF model was able to give a reasonable estimate of number of next-day discharges from the ward. Clinical staff can use this information as an aid to decisions regarding staffing and resource utilization. This foresight can also aid discharge planning such as communication and patient transfer between wards or between hospitals.

An estimate of number of free beds can also help reduce emergency department (ED) boarding time and improve patient flow [12,23]. ED boarding time is the time spent by a patient in emergency care when a bed is not available in the ward. ED boarding time severely reduces the hospital efficiency. High bed occupancy in ward directly contributes to ED overcrowding [70]. In our data, 42.81% of patients were admitted from the emergency care. An estimate of daily forecasts can be helpful in deciding the number of beds in wards to ease patient flow.

### Study Limitations

We acknowledge the following limitations in our study. First, we focused only on a single ward. However, it was a ward with different patient types, and hence the results could be an indication for all general wards. Second, we did not use patient clinical data to model discharges. This was because clinical diagnosis data were available only for 42.81% of patients who came from emergency. In a general ward, clinical coding is not done in real time. However, we believe that incorporating clinical information to model patient length of stay could improve forecasting performance. Third, we did not compare our forecasts with clinicians/managing nurses. Finally, our study is retrospective. However, we have selected prediction period separated from development period. This has eliminated possible leakage and optimism.

### Conclusion

This study set out to model patient outflow from an open ward with no real-time clinical information. We have demonstrated that using patient-level and ward-level features in modelling forecasts outperforms the traditional autoregressive methods. Our proposed models are built from commonly available data and hence could be easily extended to other wards. By supplementing patient-level clinical information when available, we believe that the forecasting accuracy of our models can be further improved.

## Abbreviations

ARIMA: autoregressive intensive moving average
ARMAX: autoregressive moving average with exogenous variables
ED: emergency department
kNN: k-nearest neighbor
MAE: mean absolute error
MAPE: mean absolute percentage error
MFE: mean forecast error
RF: random forest
RMSE: root mean square error
sMAPE: symmetric mean absolute percentage error
SVR: support vector regression
